# Benign or Malignant? Ex Vivo Confocal Laser Scanning Microscopy for Bedside Histological Assessment of Melanocytic Lesions

**DOI:** 10.3390/cancers17010151

**Published:** 2025-01-06

**Authors:** Maximilian Deußing, Lisa Buttgereit, Michaela Maurer, Alisa Swarlik, Lara Stärr, Andreas Ohlmann, Katrin Kerl-French, Michael Flaig, Elke C. Sattler, Lars E. French, Daniela Hartmann

**Affiliations:** 1 Department of Dermatology and Allergy, LMU University Hospital, LMU Munich, 80337 Munich, Germany; 2 Department of Dermatology and Allergy, Munich Municipal Hospital, 80337 Munich, Germany; 3 Department of Ophthalmology, LMU University Hospital, LMU Munich, 80337 Munich, Germany; 4 Department of Dermatology & Cutaneous Surgery, Miller School of Medicine, University of Miami, Miami, FL 33136, USA

**Keywords:** melanoma, skin tumor, fluorescence microscopy, bedside histology, dermatosurgery

## Abstract

Objective: Ex vivo confocal laser scanning microscopy (EVCM) is an emerging imaging technique, which offers rapid tissue examination. While the current literature shows promising results in the evaluation of non-melanoma skin cancer, only limited research exists on the application of EVCM in melanocytic lesions. This study aimed to assess the utility of EVCM in the characterization of melanocytic lesions and compare its findings with gold-standard histopathology. Methods: A total of 130 skin lesions, including 76 benign and 54 malignant melanocytic lesions, were prospectively collected and imaged using EVCM. Three blinded investigators were asked to identify characteristic morphologic features observed in the lesions and classify them into benign vs. malignant. The results were then compared with the corresponding histopathology. Sensitivity and specificity were calculated using contingency tables to assess the diagnostic performance. Results: The application of EVCM allowed for the visualization of cellular and tissue-level details, including cellular pleomorphism and atypical melanocytes. A comprehensive list of benign and malignant features identified by EVCM was compiled. Using these diagnostic criteria, the imaging of the inexperienced and dermatohistopathology-experienced investigator reached 67.7% concordance, and the imaging trained dermatologist obtained 69.2% agreement with dermatohistopathology in differentiating benign vs. malignant lesions. The imaging-trained dermatohistopathologist performed best with concordance up to 79.2%. Conclusions: In conclusion, EVCM is a promising technique for the rapid assessment of melanocytic lesions. Our study provides a comprehensive overview of morphologic EVCM features, which will contribute to the development of diagnostic algorithms for accurate diagnosis and appropriate treatment planning. Further studies are needed to evaluate its clinical utility and validate our diagnostic criteria.

## Simple Summary

Melanocytic lesions, such as moles and melanomas, can be challenging to diagnose accurately. While current methods like conventional histopathologic analysis are time-consuming, this study explores the use of ex vivo confocal laser scanning microscopy (EVCM), a rapid imaging technique, to examine fresh tissue immediately after surgical removal. Our goal was to identify specific morphologic features and assess how well EVCM can differentiate between benign and malignant lesions. The results showed that EVCM can provide accurate and quick diagnoses, which could help as an adjunct to conventional histopathology and speed up treatment.

## 1. Introduction

The classification of melanocytic lesions, which include benign nevi, dysplastic nevi, different subtypes of melanoma, and other malignant as well as benign melanocytic entities, poses a diagnostic challenge due to their diverse clinical presentations [1,2,3]. Accurate discrimination between benign and malignant melanocytic lesions is crucial as the early detection and treatment of melanoma significantly improve patient outcomes [4,5,6,7], often including the biopsy and assessment of adequate margins in addition to primary excision [8,9].

While conventional histopathology often is time-consuming, ex vivo confocal laser scanning microscopy (EVCM) enables the rapid examination of fresh tissue [10,11,12]. This technique offers high-resolution imaging at the cellular level, providing real-time insights that can significantly enhance diagnostic accuracy and streamline the diagnostic workflow [13,14]. EVCM has already shown promising results in the evaluation of non-melanoma skin cancer (NMSC) [15,16,17,18,19,20,21,22] and inflammatory dermatoses [23,24], whereas only limited research exists on its application in melanocytic lesions [13,25,26].

Therefore, this study aimed to evaluate the efficacy of EVCM in the characterization of melanocytic lesions. By comparing EVCM findings with traditional histopathology, we seek to identify distinctive morphologic features of benign and malignant melanocytic lesions observable through EVCM to establish a set of diagnostic criteria that can improve the differentiation of tumor dignity and potentially accelerate melanoma diagnosis.

## 2. Materials and Methods

Study participants: Between December 2022 and March 2024, 124 participants were recruited at the Department of Dermatology and Allergy of the University Hospital at Ludwig Maximilian University (LMU) in Munich. The skin types of the patients in our study ranged from Fitzpatrick type I to IV. Before being included in the study, each patient provided written informed consent, which was approved by the LMU University Hospital’s local ethics committee (Ref. No. 19-150 and Ref. No. 23-0393). In total, 130 tissue samples were obtained from patients who presented with suspected melanocytic lesions and underwent routine biopsy or excision procedures.

Ex vivo confocal laser scanning microscopy (EVCM): Immediately after biopsy or surgical excision, skin samples underwent a standardized staining protocol using ethanol (0.7 mmol/L), acridine orange (AO) (0.04 mmol/L, Sigma-Aldrich, St. Louis, MO, USA), a fluorescent dye that binds to nucleic acids to enhance the visualization of cellular structures, and FCF Fast Green (0.067 mmol/L, Sigma-Aldrich, St. Louis, MO, USA), a fluorescent dye that binds to collagen fibers followed by NaCl (0.09 mmol/L) to remove excess dye [27]. For 30 s each, every sample was completely submerged in the staining solutions, covering the entire cut surface. The Vivascope 2500 G-4 device (Vivascope, Munich, Germany) was then used to perform EVCM imaging with two laser wavelengths: 488 nm (blue) and 638 nm (red). Following this procedure, the tissue probes were positioned on object slides (R. Langenbrinck, Emmendingen, Germany), mounted using sponges and magnets, and sectioned vertically to visualize all skin layers as in standard histology [28,29,30].

Histological analysis and correlation: After imaging, the skin samples were promptly fixed in 4% buffered formalin and afterwards processed for standard paraffin embedding. All skin sections were subjected to conventional histological examination. Hematoxylin and eosin staining was employed for morphological evaluation. Where deemed necessary, the application of additional immunohistochemical staining (e.g., PRAME and Sox 10) was utilized. Histological assessments were performed by experienced dermatohistopathologists blinded to the clinical and imaging data.

EVCM image analysis: A randomized order of overview and detailed EVCM images in both digital hematoxylin–eosin (DHE) and reflectance mode (RM) [22] were displayed. Three blinded investigators were asked to describe characteristic morphologic features and analyze the cellular morphology and tissue architecture: examiner 1, an EVCM-trained and board-certified dermatohistopathologist (D.H.) with over 10 years of experience in ex vivo confocal imaging; examiner 2, an EVCM-trained dermatologist with no experience in dermatohistopathology (M.D.) but at least 3 years of experience in ex vivo confocal imaging; and examiner 3, an EVCM-unexperienced dermatologist but with 3 years of experience in dermatohistopathology (M.M.). The examiners were thus instructed to categorize the images as either “malignant” or “benign”.

In the end, a comparison between the dermatohistopathological results and the results obtained from EVCM analysis was compiled in order to ascertain the concordance and discrepancy between the two diagnostic modalities. Descriptive statistical calculations were performed using Microsoft Excel 2016 (Microsoft, Redmond, WA, USA).

## 3. Results

A total of 130 melanocytic lesions from 124 patients were included in this study. The cohort consisted of 54 females and 70 males, with an age range of 10 to 92 years (mean age: 56.0 years). In total, 76 out of 130 lesions were histopathologically classified as benign nevi, whereas 54 were graded as melanoma. Then, 51 common nevi (consisting of 13 junctional nevi, 9 dermal nevi, and 29 compound nevi), 4 blue nevi, 9 dysplastic junctional nevi, and 12 dysplastic compound nevi were classified in the benign group. The melanoma group included 8 lentigo maligna, 8 superficial spreading melanoma in situ, 12 superficial spreading melanoma, 6 nodular melanoma, 1 acral melanoma, lentiginous subtype, 6 lentigo maligna melanoma, 1 spitz melanoma, and 12 melanoma metastases (see Table 1).

**Figure 1:**
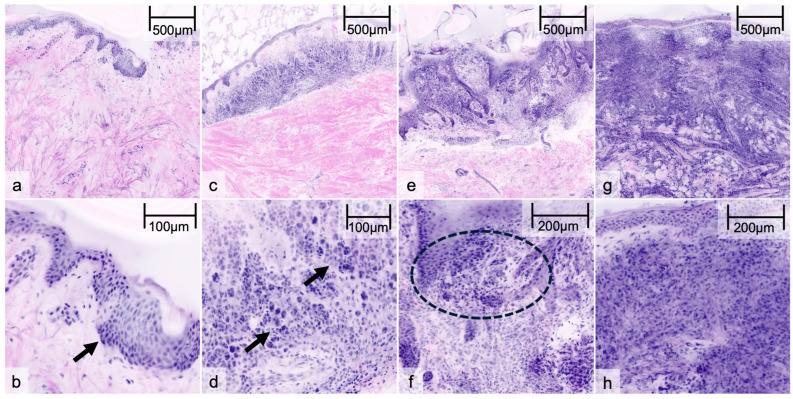
Ex vivo confocal microscopy of melanocytic lesions in digital hematoxylin–eosin: Junctional nevus (**a**) and papillomatous dermal nevus (**c**) showing well-nested melanocytic proliferations (arrows) without nuclear atypia in detailed view (**b**,**d**). Superficial spreading melanoma (**e**) and melanoma metastasis (**g**) with irregular and enlarged nests of melanocytes (circle) and cytological atypia with varying cell shapes and high mitotic activity (**f**,**h**).

EVCM provided high-resolution images in both reflectance and DHE mode, enabling the visualization of cellular and architectural features. The confocal images were analyzed and graded into benign vs. malignant independently by each examiner regarding the following morphological features (Table 2):

- Benign nevi: Characteristic features of benign nevi included well-organized nests of melanocytes, uniform cell sizes, and minimal pleomorphism. The confocal images in DHE highlighted regular, evenly distributed nucleic acid staining within the nests and no increased mitotic activity. In RM, the nests appeared as well-circumscribed, highly reflective areas (Figure 1a–d).
- Melanoma: Characteristically, melanoma images exhibited significant architectural disarray, with irregular nests and melanocytes infiltrating the dermis and epidermis. There was notable cellular pleomorphism, with varied cell sizes and shapes. The presence of atypical melanocytes with prominent nucleoli and increased mitotic figures was evident. These features were more pronounced in DHE, where the acridine orange staining provided a contrast for nuclear abnormalities. RM revealed a disrupted and heterogeneous reflective pattern consistent with malignancy (Figure 1e–h).

EVCM diagnosis was then compared to histopathological diagnosis, which was set as the gold standard. The diagnostic performance of EVCM was calculated using contingency tables. The sensitivity and specificity of EVCM in identifying malignant melanocytic lesions were as follows:

The EVCM-unexperienced and dermatohistopathology-experienced investigator (=examiner 3) was able to correctly classify 88 out of 130 lesions. In detail, 60 out of 76 skin samples were correctly graded as benign, whereas 28 out of 54 were correctly identified as malignant. This results in a sensitivity of 51.85% and a specificity of 78.95%.

The EVCM-trained dermatologist with no experience in dermatohistopathology (=examiner 2) correctly identified 90 out of 130 lesions. In total, 53 out of 76 lesions were correctly identified as benign, and 37 out of 54 malignant lesions were correctly graded as malignant. A sensitivity of 68.52% and specificity of 69.74% was obtained.

**Figure 2:**
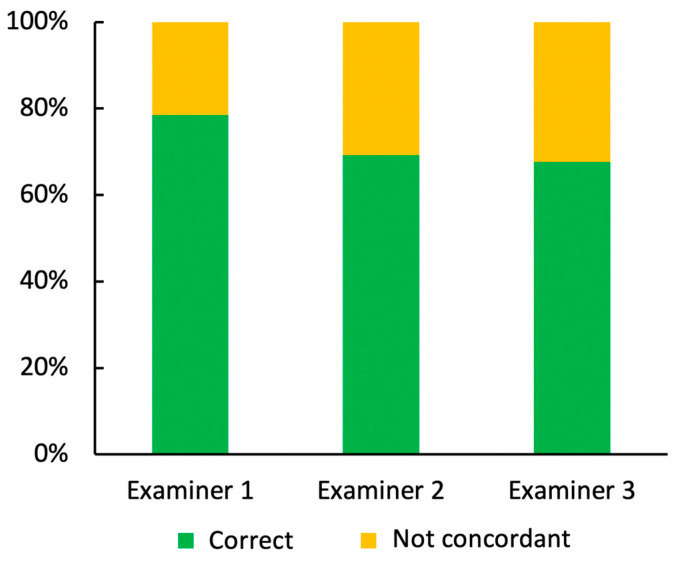
Accuracy of lesion classification between the three investigators, examiner 1 (ex vivo confocal microscopy (EVCM)-trained dermatohistopathologist), examiner 2 (EVCM-trained dermatologist with no experience in dermatohistopathology), and examiner 3 (EVCM-unexperienced and dermatohistopathology-experienced investigator).

The EVCM-trained dermatohistopathologist (=examiner 1) performed best with 103 out of 130 correctly classifications. A total of 60 out of 76 lesions were correctly classified as benign, and 43 out of 54 samples were correctly graded as malignant. The sensitivity was 79.63%, and the specificity was 78.95% (Figure 2, Table 3).

**Figure 3:**
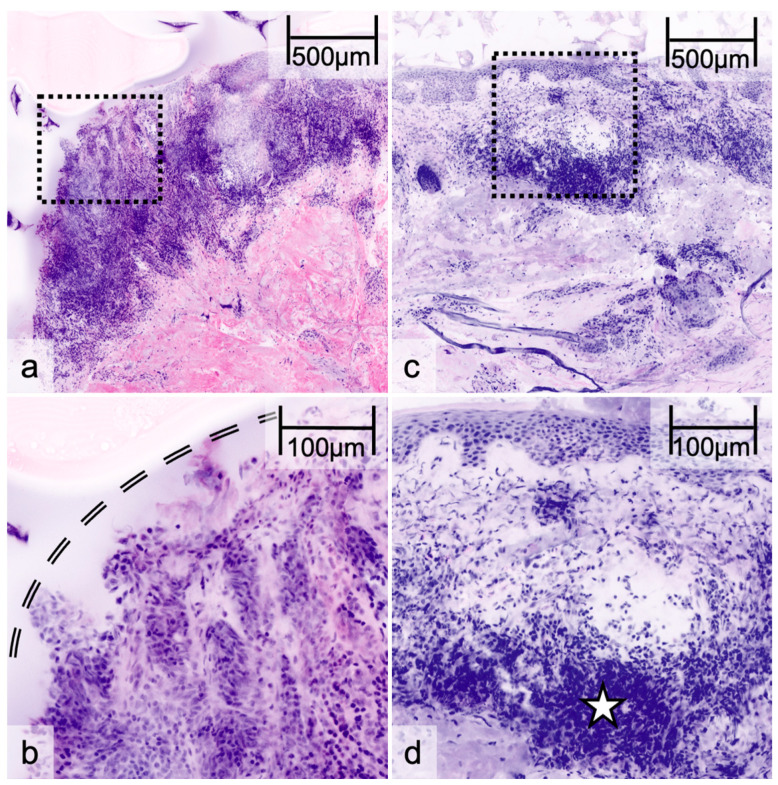
Pitfalls in ex vivo confocal microscopy: Overview and detailed images (dashed squares) showing loss of epidermis due to fixation artifact (double line) (**a**,**b**) and dermal inflammatory infiltrate (star), where analysis of single cells can be difficult (**c**,**d**).

After image evaluation, the examiners were asked to name the main difficulties that they faced while classifying the lesions. Three major issues were described, resulting in inadequate image interpretation: A subset of images was difficult to evaluate due to the inadequate visualization of the epidermis (Figure 3a,b). This included poorly recognizable cell morphology due to technical artifacts, such as those arising from difficult image preprocessing and the complex fixation of the specimen on the object slide. With 67 of 109 total errors, this was the most frequently stated error category. Another pitfall was the limited visibility of single cells, due to high reflective signals, when the lesions were containing intense pigmentation. This error category was mentioned in 25 out of 110 error cases. In a similar way, a pronounced dermal inflammatory infiltrate could lead to misdiagnosis since an inflammatory cell may sometimes mimic melanocytic cells in EVCM (Figure 3c,d). In total, 8 of 110 errors cases were referred to this category. The remaining error cases could not be classified into one of these three main categories and were based mainly on atypical image appearance in EVCM (for an overview see Table 4).

## 4. Discussion

Traditional histopathology remains the gold standard for the detailed diagnosis of melanocytic lesions. However, it involves a time-consuming process that requires tissue fixation, embedding, sectioning, and staining, which can delay diagnosis and treatment. In contrast, EVCM offers a rapid alternative that provides the real-time visualization of cellular and architectural details at microscopic level. The ability to obtain immediate results can significantly enhance clinical decision-making and patient management.

Our study demonstrated a high degree of correlation between EVCM and histopathological findings, with distinct morphological features identifiable especially in DHE. Benign nevi displayed well-organized nests of uniform melanocytes, while malignant melanoma exhibited architectural disarray, cellular pleomorphism, and increased mitotic activity. The sensitivity (examiner 1: 79.63%, examiner 2: 68.52%, and examiner 3: 51.85%) and specificity (examiner 1: 78.95%, examiner 2: 69.74%, and examiner 3: 78.95%) observed underscore the diagnostic utility of EVCM, positioning it as a valuable additional tool in the dermatological bedside assessment but also highlight the need for specialized training and expertise, which may limit its immediate applicability in all clinical settings. While EVCM was not fully effective in distinguishing benign lesions from malignant tumors, our investigators found it to be very useful in delineating melanocytic lesions from surrounding healthy tissue. This capability could also be valuable for the measurement of tumor thickness and safety margins in melanocytic lesions.

Our findings align with previous studies that have underscored the utility of EVCM in skin cancer diagnostics, though our research extends this application to melanocytic lesions specifically. However, while the results are promising, further validation is necessary to establish EVCM as a mainstream diagnostic tool. Larger, multicentric studies are required to confirm the reproducibility and reliability of our findings across diverse patient populations and lesion types. Due to the rarity of acral melanoma, lentiginous subtype, and Spitz melanoma cases among our hospital population, the number of samples available for study was quite limited. Consequently, the study was constrained to include only these samples. It is acknowledged that future studies with larger sample sizes are required to further investigate these rarer cases.

In the present study, the EVCM images were the sole focus of the examiners’ deliberate assessment. No supplementary information, such as clinical information or dermatoscopic examination results, was provided. Consequently, it cannot be excluded that the agreement with the histological results could be enhanced with the provision of additional clinical information. Further studies in clinical settings are required to ascertain this. Furthermore, the development of standardized imaging protocols and diagnostic criteria will be crucial for the widespread adoption of EVCM in clinical settings.

Diagnostic uncertainty is common with “high grade” dysplasia as melanomas and dysplastic nevi often share similar morphological features. In this instance, some melanomas were incorrectly identified as dysplastic nevi in the EVCM. It is evident that vertical visualization is particularly prone to this misdiagnosis; however, it is important to note that it is currently considered the most effective technique. Examiners also faced challenges with lesions exhibiting intense pigmentation or pronounced inflammatory infiltrates. An additional immunohistological EVCM staining with high specificity to melanocytic cells or the implementation of artificial intelligence, which may identify cellular patterns that are not recognized by the human eye, could also assist within the diagnostic workflow in the future.

## 5. Conclusions

In conclusion, our study demonstrates the potential of EVCM as an adjunct diagnostic tool for the rapid evaluation of melanocytic lesions in bedside setting. Its integration into clinical workflows may anticipate treatment decisions, improve patient outcomes, and streamline the management of melanocytic lesions. Future research should focus on larger studies and the development of standardized diagnostic criteria to facilitate the integration of EVCM into routine dermatological practice.

## Figures and Tables

**Table 1:** Overview of investigated melanocytic lesions.

Classifiation	Category	Subcategory	Number of Cases Included	Total (n = 130)
Benign	Common nevi	Junctional nevi	13	76
		Dermal nevi	9	
		Compound nevi	29	
	Blue nevi		4	
	Dysplastic junctional nevi		9	
	Dysplastic compound nevi		12	
Malignant	Lentigo maligna		8	54
	Superficial spreading melanoma in situ		8	
	Superficial spreading melanoma		12	
	Nodular melanoma		6	
	Acral melanoma, lentiginous subtype		1	
	Lentigo maligna melanoma		6	
	Spitz melanoma		1	
	Melanoma metastasis		12	

**Table 2:** Summary of characteristic morphological ex vivo confocal imaging features of benign nevi and melanoma observed in DHE (digital H&E) and RM (reflectance microscopy).

Category	Characteristic Features	DHE Findings	RM Findings
Benign nevi	- Well-organized nests of melanocytes - Uniform cell sizes - Minimal pleomorphism	- Regular, evenly distributed nucleic acid staining - No increased mitotic activity	- Well-circumscribed, highly reflective areas
Malignant melanoma	- Significant architectural disarray - Irregular nests - Melanocytes infiltrating dermis/epidermis (=pagetoid spreading) - Cellular pleomorphism with varied cell sizes/shapes - Atypical melanocytes with prominent nucleoli - Increased mitotic figures	- Pronounced nuclear abnormalities highlighted by acridine orange staining	- Disrupted, heterogeneous, and reflective pattern consistent with malignancy

**Table 3:** Correctly identified lesions for each examiner: Examiner 1 (EVCM-trained dermatohistopathologist), examiner 2 (EVCM-trained dermatologist with no experience in dermatohistopathology), and examiner 3 (EVCM-unexperienced and dermatohistopathology-experienced investigator).

	Correctly Identified Lesions in Total	Correctly Identified Benign Lesions	Correctly Identified Malignant Lesions
Examiner 1	103	60	43
Examiner 2	90	53	37
Examiner 3	88	60	28
Total number of lesions	130	76	54

**Table 4:** Distribution of errors by category in total and for each individual examiners, showing the number of error cases related to technical artifacts, pigmentation interference, and confusion with inflammatory cells.

	All Examiners	Examiner 1	Examiner 2	Examiner 3
Technical artifacts and image quality issues	67	14	26	29
Pigmentation and reflective signal interference	25	5	13	7
Confusion with inflammatory cells	8	2	3	3
Error cases in total	109	28	40	42

## Data Availability

The data presented in this study are available on request from the corresponding author. The data are not publicly available due to ethnical and privacy restrictions.
